# Dataset of genome sequence, *de novo* assembly, and functional annotation of *Ruegeria* sp. (PBVC088), a marine bacterium associated with the toxin-producing harmful dinoflagellate, *Pyrodinium bahamense* var. *compressum*

**DOI:** 10.1016/j.dib.2022.107881

**Published:** 2022-01-28

**Authors:** Grace Joy Wei Lie Chin, Salley Venda Law, Kenneth Francis Rodrigues, Jaeyres Jani, Ann Anton

**Affiliations:** a Biotechnology Research Institute, Universiti Malaysia Sabah, Jalan UMS, Kota Kinabalu, Sabah 88400, Malaysia; b Unit for Harmful Algal Bloom Studies, Borneo Marine Research Institute, Universiti Malaysia Sabah, Jalan UMS, Kota Kinabalu, Sabah 88400, Malaysia; c Faculty of Medicine and Health Science, Borneo Medical and Health Research Center, Universiti Malaysia Sabah, Jalan UMS, Kota Kinabalu, Sabah 88400, Malaysia

**Keywords:** Bacteria association, Harmful algal bloom, Illumina MiSeq, Marine bacteria, Saxitoxin

## Abstract

The dataset comprises a whole-genome sequence of *Ruegeria* sp. PBVC088, a symbiotic (Gram-negative) bacterium associated with *Pyrodinium bahamense* var. c*ompressum*, which has been associated with harmful algal blooms in the coastal waters of west Sabah, Malaysia. Harmful algal blooms contribute to economic losses for the aquaculture industry, as well as human illnesses and fatalities due to paralytic shellfish poisoning. Bacteria-algae dynamics have posited that the interaction is potentially responsible for the toxin production during a toxic harmful algal bloom event. Despite the expanding body of literature on the capabilities of these bacteria to metabolize, produce, and modify toxins autonomously, it has yet to be confirmed that these toxin-producing bacteria are capable of autonomous toxin synthesis. Saxitoxin, a paralytic shellfish poisoning toxin, is produced by a unique biosynthetic pathway, where the genetic basis for the saxitoxin production was first reported in the saxitoxin-producing cyanobacteria strain *Cylindrospermopsis raciborskii* T3 (NCBI accession no. DQ787200). The genes responsible for saxitoxin biosynthesis in dinoflagellates, have yet to be fully elucidated. The identification of cyanobacteria saxitoxin biosynthesis genes (*sxt*) may eventually lead to the identification of homologous genes within the dinoflagellates. Previous studies on the diversity of the bacterial communities associated with the same toxic *P. bahamense* harmful alga has been carried out by using both the culture-dependent 16S ribosomal RNA gene sequence analysis and culture-independent 16S metagenomic sequence analysis. This study extends the knowledge pertaining to the genomic aspect of an associated bacterium isolated from *P. bahamense* alga by adopting a whole genome sequencing approach. Here, we report the genome sequencing, *de novo* assembly, and annotation data of a bacterium, *Ruegeria* sp. PBVC088, associated with harmful alga *P. bahamense*, which can be referenced by researchers to identify the genes and pathways related to toxin biosynthesis from a much larger data set. The genome of *Ruegeria* sp. PBVC088 was sequenced using the Illumina MiSeq platform with 250 bp paired-end reads. The number of reads generated from the MiSeq sequencer was 1,135,484, with an estimated coverage of 100X. The estimated genome size for the marine bacterium was computed to be 5.78 Mb. Annotation of the genome predicted 5,689 gene sequences, which were assigned putative functions based on homology to existing protein sequences in public databases. In addition, annotation of genes related to saxitoxin biosynthesis pathway was also performed. Raw fastq reads and the final version of the genome assembly have been deposited in the National Center for Biotechnology Information (NCBI) (BioProject: PRJNA324753, WGS: LZNT00000000, SRA: SRR3646181). The genome data provided here are expected to better understand the genetic processes involved in saxitoxin biosynthesis in marine bacteria associated with dinoflagellates.

## Specifications Table

**Table:** 

Subject	Biological sciences
Specific subject area	Biotechnology, Marine Biology and Molecular Biology
Type of data	Tables and figures
How data were acquired	The whole genome sequencing was conducted on Illumina MiSeq paired-end platform.
Data format	Raw sequencing data and analyzed data
Description of data collection	Total genomic DNA extraction was performed using the DNeasy Blood and Tissue DNA Isolation Kit following manufacturer's instructions. The gDNA library was subsequently processed with the Illumina Nextera XT Library Preparation Kit following manufacturer's instructions. Paired-end sequencing of the constructed library was performed on an Illumina MiSeq (2 × 250 bp run configuration) at the Biotechnology Research Institute of Universiti Malaysia Sabah.
Data source location	The harmful algal bloom seawater samples were collected at Sepanggar Bay, Sabah, Malaysia (6.08° N, 116.12° E). The isolation of the bacterium was performed at the Biotechnology Research Institute of Universiti Malaysia Sabah.
Data accessibility	The raw sequencing data is available at BioProject, BioSample and SRA, NCBI at https://www.ncbi.nlm.nih.gov/bioproject/PRJNA324753 under the accession number of PRJNA324753 (BioProject).

## Value of the Data

• We present here the whole genome sequence data of *Ruegeria* sp., a marine bacterium associated with the saxitoxin-producing dinoflagellate, *P. bahamense* var. *compressum*.
• The data also provides researchers with a better understanding of the genes associated with the saxitoxin biosynthesis pathway in the associated *Ruegeria* bacterium.
• The data can be used by marine researchers to perform comparative genomic studies of bacteria associated with other saxitoxin-producing dinoflagellates.
• The whole genome data can be helpful as a genomic reference to facilitate future studies on the characterization of marine bacteria associated with harmful algal blooms and their contribution to the toxin biosynthesis pathway.

## 1 Data Description

**Fig. 1:**
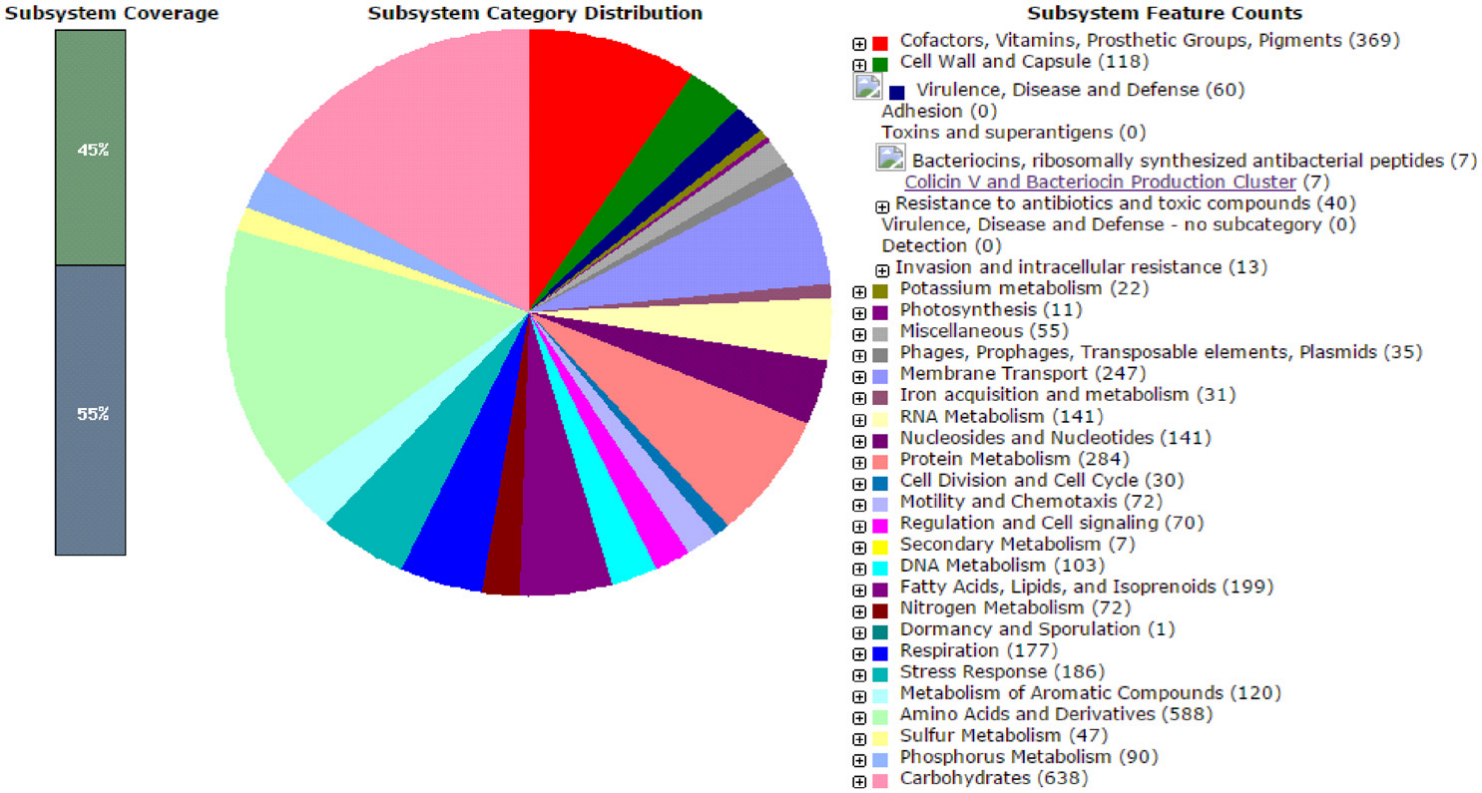
Output summary of functional distribution of protein-coding genes in the PBVC088 from RAST server.

The whole genome sequencing of a bacteria (PBVC088) associated with the harmful dinoflagellate, *P. bahamense* var. *compressum* generated approximately 1,135,484 raw pair-end reads with approximately 542.5 Mb total bases were generated (Table 1). The draft genome with the total assembly size of 5.78 Mb has 143 contigs with 64.96% GC content. The longest contig was 403,173 bp, whereas the shortest contig was 507 bp. Data generated from the MiSeq sequencer had approximately 100 times coverage, indicating that the reads were sufficient enough for a good *de novo* assembly. The contig N50 size was 149,793 bp. The N50 is the length of the average contig size and the value for this parameter was relatively high that was indicative of good contiguity of the assembly. A total of 5,635 protein coding sequences (CDSs) were identified with only 3,916 or 70 percent of the total CDSs having predicted functions and the remaining 30 percent functionally annotated as hypothetical proteins. The pie chart in Fig. 1 summarizes the functional distribution of the protein-coding genes in the PBVC088 genome, in which major genes fall within the carbohydrates (pink) and amino acids (light green) pathways.

**Table 1:** Vital statistics of the draft genome for *Ruegeria* sp. bacterium PBVC088.

Raw data	1,135,484 (542.5 M bases)
Number of contigs	143
Average length of contigs	40,437 bp
Smallest contig	507 bp
Largest contig	403,173 bp
Contig N50	149,793 bp
GC content	64.96%
Total genes	5,689
Total CDS	5,632
Number of coding genes	5,435
Number of coding CDS	5,435
Number of tRNA	49
Number of rRNA	5
Number ncRNA	3

The proposed biosynthetic pathway by Kellmann et al. [1] delineated 26 *sxt* (*sxtA* – *sxtZ*) genes with eight *sxt* proteins encoded by *sxtA, sxtG, sxtB, sxtD, sxtS, sxtU, sxtH/T*, and *sxtI*, which appeared to be directly involved in the synthesis of saxitoxin [1,2]. A total of eleven contigs (protein-coding genes) with the highest alignment score (bit score > 55) or best hit based on E-value were retrieved from the PBVC088 bacterial genome. Results of the protein similarity search have been summarized in Table 2. The remaining cyanobacterial *sxt* genes did not have any significant similarity with the protein-coding gene sequences of PBVC088. Table 3 provides a summary of shared domains between the candidate genes in PBVC088 and *sxt* genes in the cyanobacteria.

**Table 2:** BLASTP sequence similarity search of genome gainst the 26 putative *sxt* genes of STX-producing cyanobacteria, *C. raciborskii* T3 and their accesion numbers.

Genes	Candidate genes	Annotated genes	E-value	Similarity percentage (%)	Alignemnt length (base)	Putative sxt genes in C. raciborskii T3	Accession number
*sxtA*	DTG_03375	5-aminolevulinate synthase	7.00 E^−32^	29.33	358	Polyketide synthase	ABI75094
*sxtB*	DTG_00776	Cytidine deaminase	0.002	37.36	91	Cytidine deaminase	ABI175093

*sxtC - sxtD*	Unidentified						

*sxtF*	DTG_04183	Multidrug-efflux transporter	1.00 E^-37^	28.71	404	Sodium-driven multidrug and toxic compound extrusion protein	ABI75096
*sxtM*			6.00 E^−40^	31.31	444		ABI75103

*sxtG*	Unidentified						

*sxtH*	DTG_05121	3-ketosteroid-9-alpha-hydroxylase oxygenase subunit	1.00 E^−33^	27.35	340	Phenylpropionate dioxygenase	ABI75098
*sxtT*			7.00 E^−31^	26.84	339		ABI75109

*sxtI - sxtR*	Unidentified						

*sxtS*	DTG_00920	Ectoine hydroxylase	3.00 E^−06^	22.10	181	Phytanoyl-CoA dioxygenase	ABI75110
*sxtU*	DTG_01373	Sorbitol dehydrogenase	6.00 E^−42^	41.54	195	Short-chain alcohol dehydrogenase	ABI75108
*sxtV*	DTG_02991	L-aspartate oxidase	3.00 E^−17^	23.81	546	FAD-dependent succinate dehydrogenase/ fumarate reductase	ABI75107
*sxtW*	DTG_02930	Formate hydrogenlyase complex	5.00 E^−06^	31.03	58	Ferredoxin	ABI75106

*sxtX - sxtY*	Unidentified						

*sxtZ*	DTG_02593	Phosphate regulon sensor protein phoR	7.00 E^−33^	27.32	355	Histidine kinase	ABI75118

**Fig. 2:**
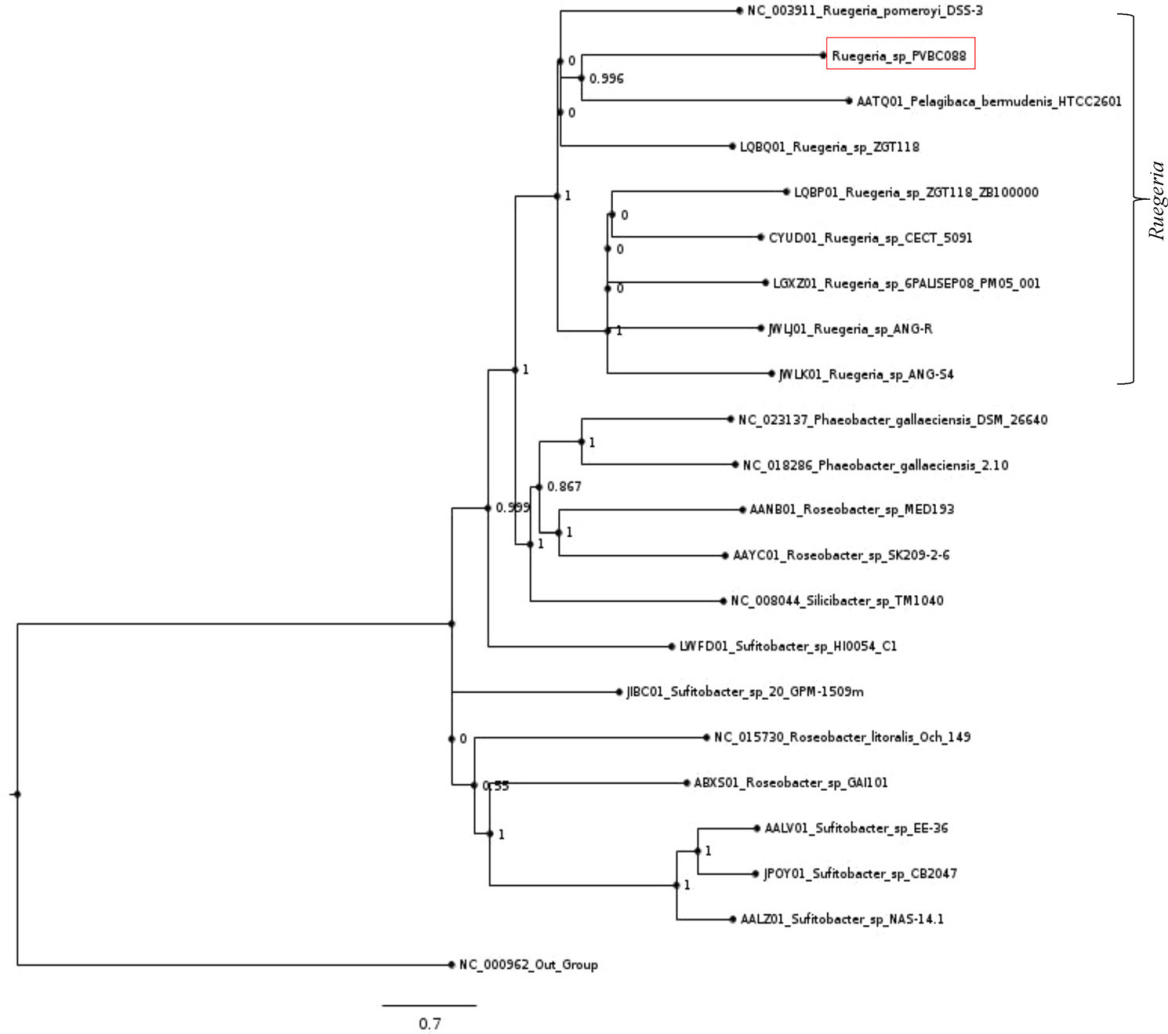
SNP phylogeny tree was constructed using parsimony algorithm-based core SNP matrix inferred from the closed genome of Roseobacter clade from NCBI database and the draft genome of *Ruegeria* sp. PBVC088 (highlighted in red box) by kSNP analysis. Bootstrap values (100 replicates) are reported above each node.

The whole genome sequence of PBVC088 was compared to a selection of closely related bacteria genomes retrieved from the Roseobacter and the NCBI databases. The phylogenetic tree is depicted in Fig. 2. The whole-genome phylogeny based on single nucleotide polymorphism (SNP) matrices resolved 21 species of the closed members of Roseobacter clade that followed the taxonomic classification of the sample, PBVC088. The kSNP analysis provided a direct visualization of the relationship among all the closed species of Roseobacter clade. All clusters have a minimum of 83% bootstrap support. The phylogeny showed that the genome PBVC088 was clustered together with the *Ruegeria* clade. Two well-defined branches were observed within the *Ruegeria* clade, where one branch contained a mixed bacteria group of *Ruegeria pomeroyi, Ruegeria* sp., and *Pelagibaca bermudenis*, whereas another branch contained only *Ruegeria* sp. bacteria. The PBVC088 was positioned within the former branch, implying high degree of similarity with *R. pomeroyi* and *P. bermudenis.* Despite the fact that *P. bermudenis* was the only distinct species within the *Ruegeria* clade, its genome may be highly similar to that of *Ruegeria* sp., indicating this organism's close association with bacteria of the genus *Ruegeria*.

In summary, we report the draft genome of *Ruegeria* sp., the first whole genome of a marine bacterium associated with the Malaysian harmful dinoflagellate, *Pyrodinium bahamense* var. *compressum*, to be sequenced, and identify genes associated with the biosynthesis of saxitoxin. Due to the limited genomic sequence resources for marine bacteria associated with toxic blooms, we believe that our research will help gain a better understanding of the biological processes that could aid in the long-term management of harmful algal blooms.

**Table 3:** Conserved domains identified in putative candidate genes by BLAST search and CD-search web service.

Genes	Candidate genes	Conserved domain
*sxtA*	DTG_03375	Cd06454, KBL_like: pyridoxal phosphate (PLP)-dependent aspartate aminotransferase superfamily (fold I); pfam00155, Aminotransferase class I and II
*sxtB*	DTG_00776	Cd01283, Cytidine deaminase
*sxtF/M*	DTG_04183	Cd13131, multidrug and toxic compound extrusion (MatE)-like protein domain
*sxtH/T*	DTG_05121	COG4638, Phenylpropionate dioxygenase
*sxtS*	DTG_00920	COG5285, Phytanoyl-CoA dioxygenase (PhyH)
*sxtU*	DTG_01373	Rossmann-fold NAD(P)(+)-binding proteins
*sxtV*	DTG_02991	pfam02910, Succinate dehydrogenase/ fumarate reductase flavoprotein C-terminal domain
*sxtW*	DTG_02930	Pfam13534, 4Fe-4S ferredoxins-type, iron sulphur binding domain
*sxtZ*	DTG_02593	Cd00075, Histidine kinase-like ATPases Cd00082, Histidine kinase A

## 2 Experimental Design, Materials and Methods

### 2.1 Study area

Seawater samples containing the toxic marine dinoflagellate, *P. bahamense* var. *compressum* was collected from Sepanggar Bay (6.08° N, 116.12° E) in December of 2012 during the period at which high concentrations of paralytic shellfish poisoning toxin was detected in the coastal waters of Sabah [3].

### 2.2 Sample preparation

The PBVC088 bacterium was isolated from the clonal culture of the harmful alga *P. bahamense* var. *compressum* (CC-UHABS-040(M)) following the previously described method [4]. Early, late exponential, and stationary growth phases of the microalgal cultures were serially diluted (10-fold dilution) in sterile seawater. The isolated bacterium was maintained on sterile marine agar (Difco) at 37 °C. To obtain pure culture, bacteria from a dilution containing 50 to 100 colonies were isolated from one replicate plates and replated separately onto the marine agar. For DNA extraction, the PBVC088 isolate was cultured overnight at 37 °C in 5 ml of marine medium (Difco) in an incubator shaker. A total of 1 ml of bacterial cell culture was pelleted in a 1.5 ml centrifuge tube by centrifugation at 5000 x g for 10 min. The genomic DNA of the pelleted bacterial cell culture was extracted using the DNeasy Blood and Tissue DNA Isolation Kit (Qiagen Biotechnology) following the manufacturer's instructions. The concentration and quality of the extracted DNA were assessed using the Qubit 2.0 fluorometer (Life Technologies Corporation) and Nanovue Plus Spectrophotometer (GE Healthcare), respectively, before proceeding to library preparation.

### 2.3 Genome sequencing, assembly and annotation

Next generation sequencing library preparations were constructed following instructions listed in Illumina Nextera XT Library Preparation Kit. The prepared library was then loaded onto an Illumina MiSeq system (Illumina, USA) according to the manufacturer's instructions. Sequencing was carried out using 250 paired-end configurations; base calling and image analysis were conducted by the MiSeq Control Software on the MiSeq instrument. The obtained raw sequences were filtered based on quality using Fastq Quality Filter in Fastx Toolkit [5]. The reads were then subjected to adapter sequence removal and low-quality region or reads trimming using Scythe v0.994 [6] and Trimmomatic v0.35 [7] for quality trimming of reads based on quality threshold of Q-score 25. The draft genome was *de novo* assembled using Iterative De Brujin Graph De Novo Assembler IDBA-UD software [8]. The assembler is suitable for short reads sequencing data with highly uneven sequencing depths. The assembled reads were annotated using rapid prokaryotic genome annotation (PROKKA) [9] and Rapid Annotation using Subsystem Technology (RAST) server version 2.0 [10].

### 2.4 Analyses of genes putatively involved in saxitoxin biosynthesis

The protein sequences *(faa*. files) that were predicted from the nucleotide assemblies were subjected to *sxt* genes identification and analysis. Saxitoxin (STX)-related genes were identified through sequence similarity searches using BLASTP (expectation value, E-value < 1e^-5^) against the 26 putative *sxt* genes in the toxic cyanobacterium *C. raciborskii* T3 [1] as queries. Candidate genes resulted from the protein similarity search was then analyzed against the Conserved Domain Database (CDD) using CD-search web service [11].

### 2.5 Whole genome sequence phylogenetic analysis

The core-SNP was determined using kSNP3 (alignment-free sequence analysis method) [12]. Single nucleotide polymorphisms (SNPs)-based phylogenetic tree was performed using the maximum likelihood method in MEGA6 (Molecular Evolutionary Genetic Analysis) Software [13].

## CRediT authorship contribution statement

**Grace Joy Wei Lie Chin:** Conceptualization, Funding acquisition, Project administration, Methodology, Supervision, Validation, Writing – original draft. **Salley Venda Law:** Resources, Investigation, Software, Formal analysis, Data curation, Validation. **Kenneth Francis Rodrigues:** Funding acquisition, Methodology, Supervision, Validation, Writing – review & editing. **Jaeyres Jani:** Software, Formal analysis, Data curation, Writing – review & editing. **Ann Anton:** Conceptualization, Resources, Supervision, Visualization.

## Declaration of Competing Interest

The authors declare that they have no known competing financial interests or personal relationships that could have appeared to influence the work reported in this paper.
